# A prospect of cell immortalization combined with matrix microenvironmental optimization strategy for tissue engineering and regeneration

**DOI:** 10.1186/s13578-018-0264-9

**Published:** 2019-01-05

**Authors:** Yiming Wang, Song Chen, Zuoqin Yan, Ming Pei

**Affiliations:** 1 Stem Cell and Tissue Engineering Laboratory, Department of Orthopaedics, West Virginia University, PO Box 9196, 64 Medical Center Drive, Morgantown, WV 26506-9196 USA; 2 Department of Orthopaedics, Zhongshan Hospital of Fudan University, 180 Fenglin Road, Shanghai, 200032 China; 3 Department of Orthopaedics, Chengdu Military General Hospital, Chengdu, 610083 Sichuan China; 4 WVU Cancer Institute, Robert C. Byrd Health Sciences Center, West Virginia University, Morgantown, WV 26506 USA

**Keywords:** Cell senescence, Decellularized cell-deposited extracellular matrix, Differentiation, Immortalization, Proliferation, SV40, Tissue engineering

## Abstract

Cellular senescence is a major hurdle for primary cell-based tissue engineering and regenerative medicine. Telomere erosion, oxidative stress, the expression of oncogenes and the loss of tumor suppressor genes all may account for the cellular senescence process with the involvement of various signaling pathways. To establish immortalized cell lines for research and clinical use, strategies have been applied including internal genomic or external matrix microenvironment modification. Considering the potential risks of malignant transformation and tumorigenesis of genetic manipulation, environmental modification methods, especially the decellularized cell-deposited extracellular matrix (dECM)-based preconditioning strategy, appear to be promising for tissue engineering-aimed cell immortalization. Due to few review articles focusing on this topic, this review provides a summary of cell senescence and immortalization and discusses advantages and limitations of tissue engineering and regeneration with the use of immortalized cells as well as a potential rejuvenation strategy through combination with the dECM approach.

## Background

Tissue and organ failure is a prominent health issue that cannot be ignored. Surgical intervention, organ transplantation, artificial substitutes and mechanical devices are methods to address this issue but all have undesirable short- and long-term consequences [1]. Tissue engineering is an attractive method that enables fabrication of functional tissue for tissue regeneration as well as the establishment of physiological and pathological models for mechanistic studies [2]. This technique can harness the intrinsic regenerative potential of primary cells and expand them in a controlled environment before reintroduction into the patient’s body. These natural, synthetic or semisynthetic tissue and organ mimics are expected to function normally in a tissue-specific pattern as required [1, 3]. However, primary cells derived from non-cancerous tissues have a finite lifespan and decreased proliferation ability when cultured in vitro. After a limited number of divisions, cells enter a viable state of permanent quiescence, termed cellular senescence [4]. Cellular senescence, regulated by both intrinsic and extrinsic factors, is characterized as two key phenotypes, a stable proliferation arrest and altered secretory pathway, the senescence-associated secretory phenotype (SASP) [5].

**Fig. 1:**
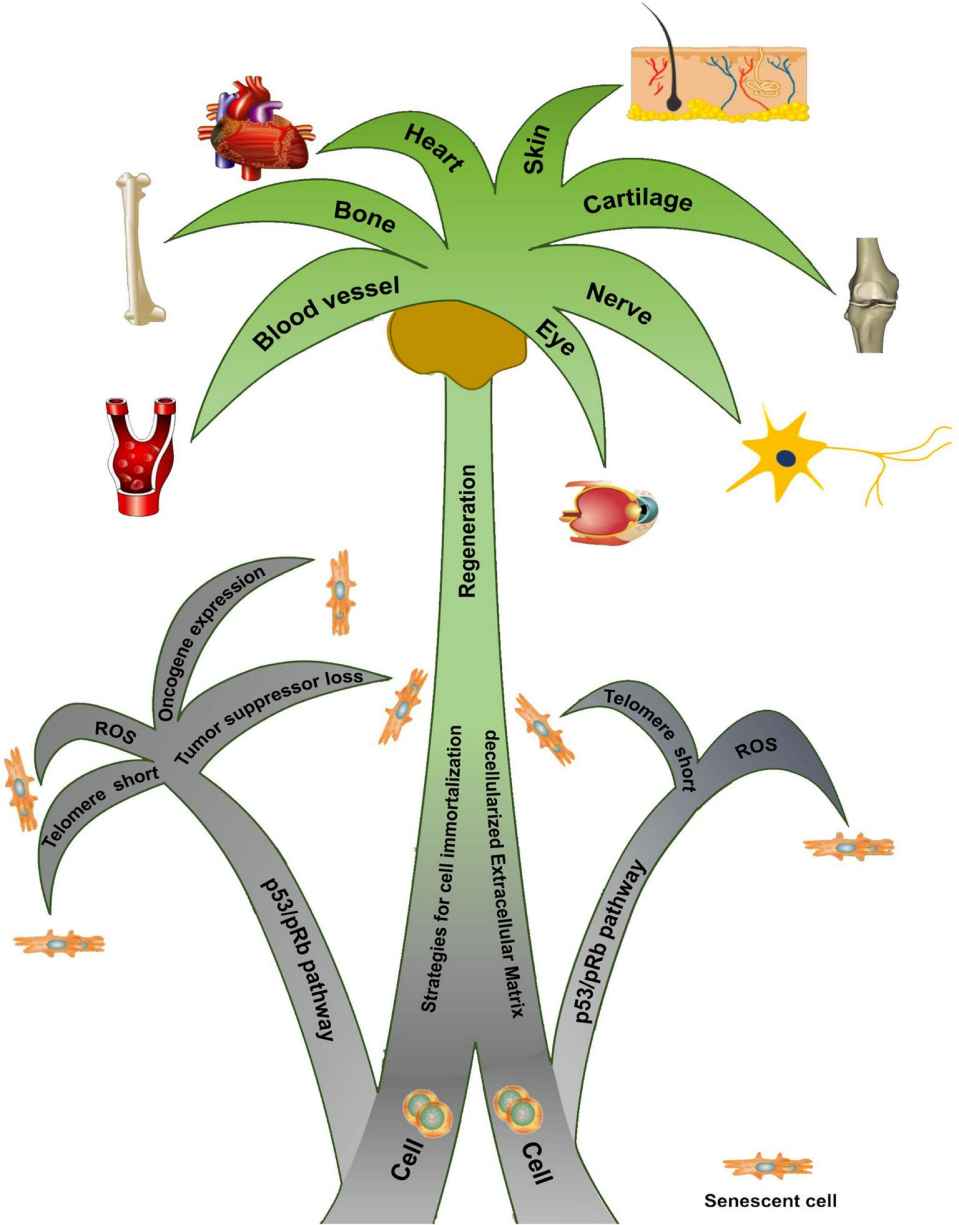
A schematic diagram of immortalization strategy combined with the decellularized cell-deposited extracellular matrix approach to overcome cell senescence and promote tissue regeneration

In order to acquire an abundant number of cells for functional tissue engineering, cellular senescence is the major obstacle that needs to be overcome. Numerous attempts have been made in past decades to deal with cellular senescence in order to achieve successful immortalization of primary cells. To establish immortalized cell lines for research and clinical use, strategies have been applied including internal genomic or external matrix microenvironment modification. Considering the potential risks of malignant transformation and tumorigenesis of genetic manipulation, environmental modification methods, especially the decellularized cell-deposited extracellular matrix (dECM)-based preconditioning strategy, appear to be promising for tissue engineering-aimed cell immortalization. Due to few review articles focusing on this topic, this review provides a summary of cell senescence and immortalization and discusses advantages and limitations of tissue engineering and regeneration with the use of immortalized cells as well as a potential rejuvenation strategy when combined with the dECM approach (Fig. 1).

## Cellular senescence

Cellular senescence is a process that imposes irreversible proliferative arrest on cells in response to internal and external environmental changes. Various stressors, including progressive telomere erosion, oxidative stress, the expression of oncogenes and the loss of tumor suppressors, contribute to the occurrence of cellular senescence.

### Replicative senescence

As vital structures that cap and protect the ends of linear chromosomes [6], shortening of telomeres happens at every fission which eventually causes cells to reach their “Hayflick limit” which halts growth after approximately 60 population doublings [4, 7]. When telomeres are too short to function normally for capping, replicative senescence (M1 stage, a cellular growth arrest) occurs [8–10]. The critically short telomeres are detected by cells as double-strand breaks and trigger a deoxyribonucleic acid (DNA) damage response (DDR) that consists of a series of signaling events centered on two anti-proliferative mechanisms, the p53/p21 and p16/tumor suppressor retinoblastoma protein (Rb) pathways. This cessation allows cells to repair the DNA damage, but if the damage continues to exceed a certain limit, apoptosis or senescence may occur [11]. Regulated by its upstream partner p16, one of the cyclin-dependent kinase inhibitors (CKIs), Rb controls cell cycle progression from G_1_ into S phase by binding to and suppressing the activity of E2F transcription factor 1 (E2F1) [10, 12, 13].

Many researchers have verified the importance of both pathways in directing senescence as the suppression of either p53 or Rb alone failed to achieve cell immortalization [14–17]. However, there were still reports showing that, in human mammary epithelial cells and mesenchymal stem cells (MSCs), inactivation of p16 alone allows human cells to avoid senescence [18, 19]. Meanwhile, in human diploid fibroblasts, the p53 mutant alone is able to suppress cellular senescence [20]. These findings raise the possibility that these two pathways may function differently among different cell strains.

However, abrogation of the p53/p21 and p16/Rb pathways will only lead to a “pre-immortal” state instead of an “immortal” status for cells. Terminal telomere shortening still exists and will eventually lead to the M2 stage, characterized as massive cell death [10, 15, 21]. In most cases, the stabilization of telomeres is achieved through the introduction of telomerase, an enzyme that synthesizes telomeric repeats and adds them to the ends of chromosomes for the compensation of inevitable loss with each round of DNA replication [22].

### Premature senescence

Senescence also happens in conditions that are not dependent on telomere erosion or dysfunction. This process is often referred to as “premature” since it can arrest growth prior to reaching the “Hayflick limit” [23]. Various conditions have been identified that may result in premature cellular senescence.

### Stress-induced senescence

During a long-term in vitro cell expansion, laboratory culture conditions, generally defined as a lack of surrounding cell types and support from extracellular matrix (ECM), abnormal growth factors and oxygen (O_2_) level, expose the cell to excessive oxidative stress and induce oxidant production [24–27]. The excessive levels of reactive oxygen species (ROS), including hydrogen peroxide (H_2_O_2_), hydroxyl radical (OH^−^) and superoxide anion (O_2_^−^), are detectable during long-term culturing of MSCs, accounting for stress-induced senescence [24–26]. H_2_O_2_ could directly affect cellular DNA, trigger DDR and subsequent p16/Rb and p53 pathways, leading to cell cycle arrest [28–32].

### Oncogene-induced senescence

There is accumulating evidence showing both in vitro [33–35] and in vivo [36, 37] oncogene activation, including *Ras*, *Raf*, *BRAF* (human gene that encodes a protein called B-Raf) and *E2F1*, can cause an irreversible cell growth arrest, termed oncogene-induced senescence. In normal primary cells, *Ras* activation leads to compulsory replication, triggering DDR and the subsequent senescence-based pathways [33, 38, 39]. *Raf* encodes proteins that function as a downstream effector of the Ras family and activate the extracellular signal-regulated kinase (MAPK) kinase (MEK) in cascade, which in turn, activates extracellular signal regulated kinase 1/2 (ERK1/2) [40]. Interestingly, Raf itself is able to elicit senescence in IMR-90 cells [34]. The p16/Rb and p53 pathways are crucial mediators of oncogene-induced senescence; however, the p16/Rb pathway in oncogene-induced senescence acts differently than in replicative senescence [33, 41, 42]. The *BRAF* gene, a downstream effector of Ras, is an intracellular effector of the MAPK signaling cascade that facilitates transmembrane signal transduction [43]. In primary cells, the expression of *BRAF*^V600E^ is known to induce transient stimulation of proliferation and subsequently trigger cellular senescence as demonstrated in normal cells including melanocytes [44], fibroblasts [45] and stem cells [46]. E2F1 is the founding member of the E2F family, a regulatory protein that drives cell cycle progression through interaction with Rb [47]. When a cell prepares to enter the S phase of the cell cycle, E2F1 is released from the Rb-E2F1 complex, activating the downstream target genes regulating normal entry into S phase [48]. Interestingly, in normal human fibroblasts, E2F1 and its target gene p14 (ARF) are responsible for the induction of cellular senescence [35]. Meanwhile, E2F1 knock-out mouse embryonic fibroblasts demonstrated attenuated senescence and ROS levels [49].

### Tumor suppressor loss-induced senescence

Tumor suppressors are the counterpart of oncogenes, and their loss can elicit cellular senescence. Depletion of *NF1* (Neurofibromatosis 1), a tumor suppressor gene, induces senescence in human fibroblasts [50]. Similarly, loss of *BTG3* (B-cell translocation gene 3), a member of the anti-proliferative BTG gene family and a downstream target of p53, triggers cellular senescence as well [51]. Inactivation of *VHL* (von Hippel-Lindau tumor suppressor) induces an efficient senescence in mouse fibroblasts and primary renal epithelial cells under atmospheric conditions (21% O_2_); however, loss of *VHL* only causes a decreased cell proliferation instead of cell arrest in human renal epithelial cells [52, 53]. Similarly, acute loss of tumor suppressor gene *PTEN* (phosphatase and tensin homolog) induces growth arrest through the p53-dependent cellular senescence pathway in mouse prostate both in vitro and in vivo whereas, in systemic lupus erythematosus patients, the complete loss is significantly related to advanced cancer and poor outcomes [54–56]. These findings raise the possibility that tumor suppressors may function differently according to different species and cell types.

## Signaling pathways involved in cellular senescence

**Fig. 2:**
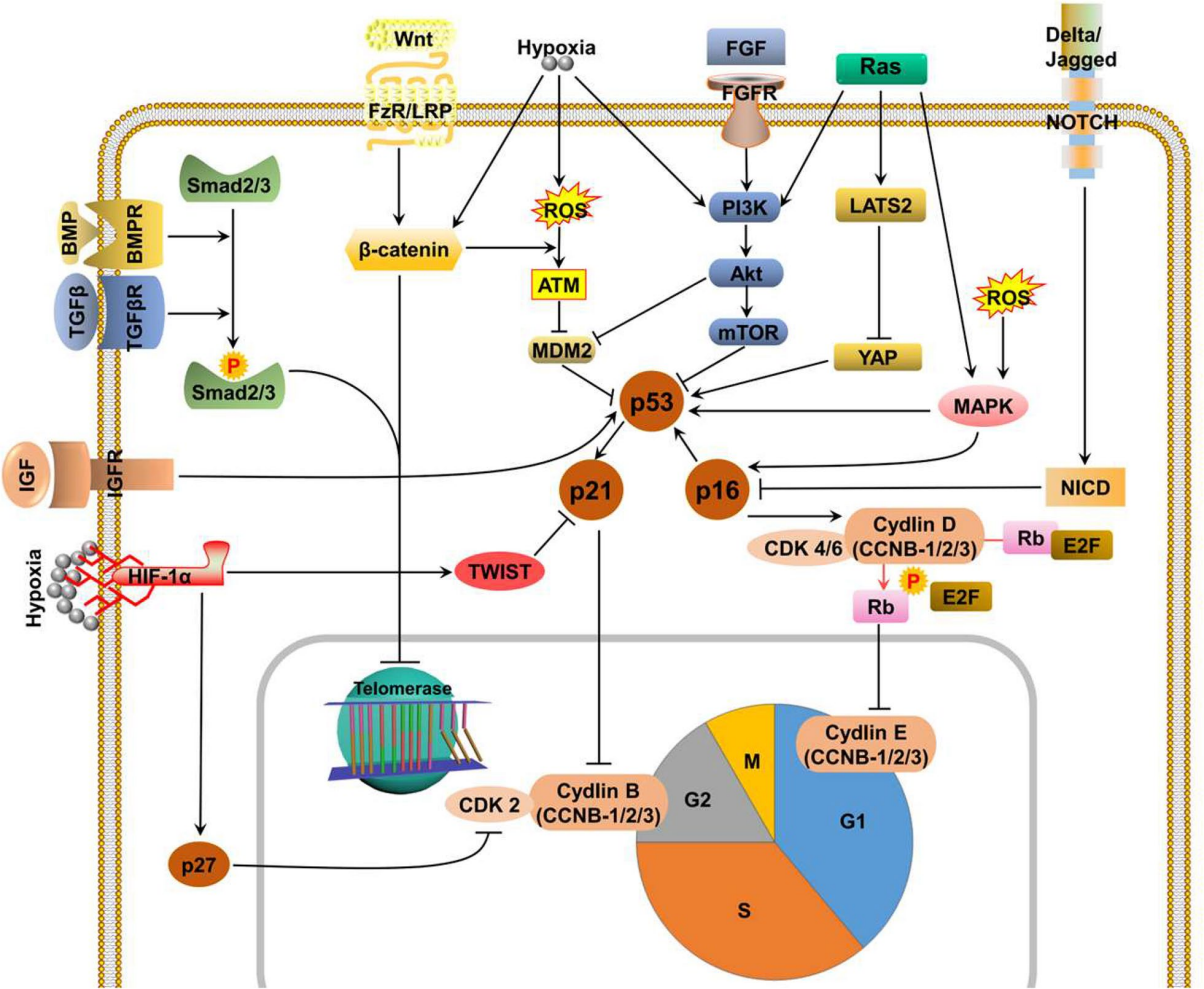
Signaling pathways mediating the cellular senescence process. In response to telomere erosion, ROS production, the expression of oncogenes and the loss of tumor suppressors, various signaling pathways including TGFβ, BMP, Wnt, MAPK, FGF, IGF, HIF and Hippo pathways are all actively involved in cell cycle regulation, which eventually influences the cellular senescence process of primary cells

Despite the abovementioned p53/p21 and p16/Rb pathways, other signaling pathways are also involved in cellular senescence, including, but not limited to, transforming growth factor β (TGFβ)/bone morphogenetic protein (BMP), Wingless/Int (Wnt)/β-catenin, MAPK, phosphatidylinostitide 3 kinase (PI3K)/protein kinase B (AKT)/mammalian target of rapamycin (mTOR), Hippo, NOTCH, fibroblast growth factor (FGF) and insulin-like growth factor (IGF) and hypoxia inducible factor (HIF) (Fig. 2).

### TGFβ/BMP signaling pathways

TGFβ is a classic regulator for chondrogenic differentiation but its role in cell expansion remains controversial [57, 58]. TGFβ activation is positively involved in the induction of cellular senescence of all kinds of species [59–61]. In human breast cancer cells, TGFβ negatively mediates telomerase activity through its downstream effector, Smad3 [62, 63]. For stress-induced senescence, TGFβ contributes to ROS production and activation of DDR during the senescence of human fibroblasts and bone marrow-derived MSCs (BMSCs) [64, 65]. The kinase ataxia-telangiectasia mutated (ATM) is a key player in nuclear DDR [66]. Meanwhile, TGFβ is required for oncogene-induced senescence that is independent of the p16/Rb and p53 pathways; attenuation of TGFβ inhibits premature senescence in human mammary epithelial cells [67, 68].

BMPs are secreted signal factors belonging to the TGFβ superfamily and are involved in embryonic development and cellular processes [69]. Similar to the function of TGFβ, BMP receptor II/Smad3 contributes to telomerase inhibition and telomere shortening in human breast cancer cells, leading to replicative senescence [70]. Similar results were observed in primary cells as the BMP signaling axis plays an important role in oncogene-induced senescence of mouse fibroblasts [71].

### Wnt/β-catenin pathway

Wnts are highly conservative proteins that participate in embryonic development and homeostatic mechanisms in adult tissues [72]. Wnt signals appear to be an important regulator of both premature senescence and replicative senescence. On one hand, the Wnt/β-catenin signaling pathway interacts with the p53/p21 pathway for ROS production to induce MSC senescence [73–76]. On the other hand, Wnt3a/β-catenin also plays a critical role in hedging replicative senescence of MSCs, probably through regulation of a telomerase subunit—telomerase reverse transcriptase (TERT) [72, 77]. Meanwhile, Wnt/β-catenin signaling enhances rat nucleus pulposus cell senescence as well as induces the expression of TGFβ, another strong promoter of cellular senescence [78].

### MAPK pathway

The MAPK signaling cascade, mainly including ERK, c-Jun N-terminal kinase (JNK) and p38, regulates several physiological and pathological processes [40]. p38 is well-recognized to be involved in premature cellular senescence [79, 80]. The major role of the p38 pathway in oncogene-induced senescence is induced by the oncogene Ras or its downstream effector, Raf-1 [81, 82]. Ras provokes premature senescence through activation of the MEK/ERK pathway, followed by p38 activation [81]. Shin and colleagues found that ERK2 is responsible for Ras-induced senescence in mouse embryonic fibroblasts [83]. Despite premature senescence, ERK is also actively involved in replicative senescence and suppression of ERK signaling rescues cardiac progenitor cells from replicative senescence when expanded in vitro [84]. The JNK signal was reported to be active in responding to a wide range of DNA-damaging agents from both endogenous and exogenous causes and JNK phosphorylation is involved in senescence-associated matrix metalloproteinase-1 production in response to ROS in IMPR-90 cells [85–89]. However, the senescence-promoting role of JNK was challenged as it was also revealed to antagonize p38-induced p16 activation [90]. Moreover, JNK acts as a negative regulator of p53 tumor suppressor to suppress p53-dependent senescence in mouse embryonic fibroblasts [91]. An increase of intracellular ROS levels can suppress the growth of cancer cells and induce cellular apoptosis by mediating MAPK signaling components [92].

### PI3K/AKT/mTOR pathways

PI3Ks and their downstream mediators AKT and mTOR constitute the core component of the PI3K/AKT/mTOR signaling pathway which is precisely controlled under normal physiological conditions and is a frequently hyperactivated pathway in cancer [93]. Similar to the MEK-ERK pathway, PI3K is one of the main downstream effectors of Ras dependent signaling and its activation plays dual roles in cell cycle regulation as it can promote cell cycle progression as well as cause cell cycle arrest [94]. Recent studies reveal the involvement of PI3K/AKT/mTOR in the regulation of replicative senescence in human vascular smooth muscle cells [95]. Moreover, a constitutively active, myristoylated form of AKT leads to oncogene-induced senescence in primary cultured human endothelial cells and murine fibroblasts; the loss of PTEN triggers senescence through activation of the PI3K/AKT pathway in mouse prostate [56, 96]. However, a recent report subverted the positive role of the PI3K/AKT/mTOR pathway in senescence-induction by introducing the fact that activation of this pathway abolished BRAF^V600E^-induced senescence in both primary human fibroblasts and primary human melanocytes [97]. Moreover, targets of the PI3K/AKT signaling pathway have been found to promote cell survival [98] and the activation of the PI3K/AKT pathway can be induced by TGFβ, leading to a pro-survival/anti-apoptotic effect in both human nasopharyngeal carcinoma cells [99] and mesenchymal cells/fibroblasts [100].

### Hippo pathway

The Hippo pathway is a tumor suppressor pathway; dysregulation of this pathway can lead to uncontrolled cell proliferation and tumorigenesis [101]. Yes-associated protein 1 (YAP), a major downstream effector of the Hippo pathway, is phosphorylated and inactivated by the serine/threonine kinases large tumor suppressor 1 (LATS1) and LATS2 [102, 103]. YAP dephosphorylation is associated with the senescence of rat nucleus pulposus cells and overexpression of YAP in primary human keratinocytes blocks clonal evolution and induces cell immortalization [104, 105]. Coordination of the Hippo pathway and p53 occurs in response to various types of stress signals including replication and oncogenic Ras. LATS2 cooperates with p53 to induce p21 expression, resulting in cellular senescence [106]. LATS2 also plays an important role in the oncogenic H-Ras induced stress checkpoint in a p53-dependent pathway [107].

### Notch pathway

The Notch pathway is an evolutionarily highly conserved signaling pathway that is associated with a variety of cellular processes including cell-fate determination, proliferation and death. In mammals, the Notch family has five ligands and four receptors [108, 109]. There is accumulating evidence that abnormal Notch signaling has been implicated in multiple facets of cancer biology, and Notch can behave as either an oncogene or a tumor suppressor depending on cell context [110, 111]. The oncogenic function of Notch has been demonstrated in several types of cancer including melanoma [112], breast cancer [113] and brain tumors [114]. Activated Notch 1 significantly enhances the rate of glycolysis, which prevents cellular senescence of human adipose-derived stromal cells (ADSCs) through HIF1 activation and p53 inactivation [115]. On the other hand, the Notch pathway is found to serve as a tumor suppressor in the progression of carcinoma including bladder cancer [116], medullary thyroid carcinoma [117] and pancreatic cancer [118]. Enforced Notch activation in human endothelial cells is associated with cellular senescence with the involvement of p16 [119]. Down-regulation of Notch 3 in human fibroblasts and mammary epithelial cells delays the onset of senescence and extends cell lifespan [120, 121].

### FGF and IGF pathways

FGFs are well-recognized for their critical roles in embryonic development [122]. The mitogenic effect of FGF has been demonstrated by promoting proliferation while maintaining stemness of MSCs in vitro [123–125]. FGF2 treatment led to an early increase in telomere size in MSCs, probably due to its ability to increase TERT mRNA expression [126, 127]. FGF signals negatively regulated MSC senescence through interaction with PI3K/AKT/MDM2 (mouse double minute-2 homolog) in the mouse and through down-regulation of TGFβ expression in human MSCs [128, 129]. Surprisingly, FGF23 can also induce premature senescence in human MSCs from skeletal muscle via the p53/p21 oxidative-stress pathway [130].

IGFs are considered detrimental to cell survival due to their role in diminishing tissue resistance to oxidative stress and shortening lifespan [131, 132]. In mouse, rat and human primary vascular smooth muscle cells, IGFI induces cellular senescence dependent on the upregulation of p53 [133]. Additional evidence has revealed that IGF binding protein-5 is upregulated in the regulation of premature senescence of umbilical vein endothelial cells through a p53-controlled mechanism [134, 135]. These findings may be due to the mechanism whereby IGFI is capable of inducing telomere shortening [136]. However, opposite results were found in human annulus fibrosus cells as IGFI alleviates cellular senescence [137]. In this scenario, the regulatory roles of both FGFs and IGFs relating to cellular senescence are context-dependent.

### HIF pathway

HIFs are composed of two different basic-helix-loop-helix-PAS transcription factors, HIF1α and HIF1β [138]. It has been proposed that a classic cellular response to hypoxia is cell cycle arrest at the G_1_/S interface through the regulation of p27 expression in which HIF1α is a major mediator [139, 140]. On the other hand, HIFs are involved in the promotion of cancer growth and the loss of HIFs induces the production of ROS and the activation of proteins p53 and p16 [141, 142]. HIF1α is involved in the suppression of senescence through regulation of p53 and p21 in human diploid fibroblasts [143]. When expanded in hypoxic conditions, human MSCs and the old human endothelial progenitor cells escape senescence through regulation of HIF1α-TWIST-p21 axis [144, 145]. Moreover, hypoxia led to PI3K/AKT pathway activation and elevated expression of Wnt coreceptor low-density lipoprotein receptor-related protein 5 (LRP5), contributing to the promotion of self-renewal and decreased cellular senescence of marrow-isolated adult multilineage inducible cells [146].

## Immortalization of cells through genetic modification

To achieve cell lifespan extension, biotechnological methods are often used for direct manipulation of a cell’s genome. However, concerns still exist regarding genomic stability and tumorigenicity after genetic modification. For MSCs and progenitor cells, the potential loss of differentiation ability after genetic modification is a problem that cannot be overlooked.

### Genetic modification

The introduction of viral oncogenes/oncoproteins and TERT are two typical methods for this type of genetic modification (Table 1).

**Table 1:** Immortalization of primary cells for therapeutics and research

Immortalization	Cell type		References
Oncoprotein(s)	Human	Articular chondrocytes, bone marrow endothelial cells, cranial suture progenitors, foreskin keratinocytes, hepatocyte, keratinocytes, liver renal proximal tubular epithelial cells, mammary epithelial cells, marrow stromal cells, nucleus pulposus cells, podocyte cells, sinusoidal endothelial cells, umbilical cord blood endothelial progenitor cells, umbilical vein endothelial cells, uterine cervix epithelial cells	[154, 233, 238, 287–303]
	Animal	Mouse articular chondrocytes, rat renal proximal tubular epithelial cells	[304]
Oncogene(s)	Human	Prostate epithelial cells, neural precursors, embryonic stem cell-derived MSCs	[160, 161, 305]
Oncoprotein(s) and oncogene(s)	Human	Embryonic fibroblasts, keratinocytes	[162, 306]
TERT	Human	Adipose-derived stromal cells, amnion-derived stem cells, bone marrow-derived MSCs, cementum-lining cells, cord blood MSCs, dermal microvascular endothelial cells, embryonic stem cells, fetal hepatocytes, hepatic stellate scavenger cells, neural progenitor cells, osteoblasts, periodontal ligament progenitor cells, renal proximal tubule epithelial cells, vocal fold fibroblasts	[178, 215, 307–318]
	Animal	Mouse temporomandibular joint disc cells	[319]
TERT and oncoprotein(s)	Human	Adipose-derived stromal cells, amniotic fluid-derived mesenchymal stem cells, bone marrow-derived MSCs, ovarian surface epithelial cells, pancreatic β cells, pancreatic islet cells, periodontal ligament fibroblasts, pulmonary microvascular endothelial cells, renal proximal tubule epithelial cells	[244, 247, 320–328]
	Animal	Rat ventricular cardiomyocytes	[329]
TERT and oncogene(s)	Human	Fetal pancreatic epithelial cells, placenta-derived MSCs, adipose-derived stromal cells	[246, 330–332]
	Animal	Bovine germ line stem cells	[163]
TERT, oncoprotein(s) and oncogene(s)	Human	Bone marrow-derived MSCs	[333]
TERT and suppression of p53 or Rb pathway	Human	Mammary epithelial cells, ovarian surface epithelial cells	[176, 334, 335]
TERT and cyclin-dependent kinase 4	Human	Bronchial epithelial cells	[336]
Mutant p53	Human	Mammary epithelial cells	[337, 338]
Irradiation and oxidative stress	Human	Mammary epithelial cells	[339, 340]
Chemical carcinogens	Human	Mammary epithelial cells	[341]
	Animal	Syrian hamster dermal fibroblasts and embryo cells, rat hepatocytes	[342–344]
TERT and cytotoxic T lymphocyte-associated antigen 4-Ig	Human	Bone marrow-derived MSCs	[345]

#### Viral oncogenes/oncoproteins

Viral oncogenes that are able to inactivate both pRb and p53 can overcome M1 (a barrier in which normal cells senesce and cease replication) and significantly prolong cell lifespan. For several decades, simian virus 40 (SV40) early region genes have been commonly used for cell immortalization and cell line establishment [147, 148]. SV40 is limited to two proteins as the large T (LT) and small t antigen (ST). LT is mainly responsible for the SV40-extended lifespan based on its ability to interact with growth suppressors—pRb and p53. LT binds to the pRb-E2F complex via its pocket binding site including AA101–118 and the J domain that acts as a chaperone, leading to the dissociation of E2F from the LT-pRb complex [149]. Meanwhile, LT binds to p53 therefore suppressing the p53 pathway [150]. More interestingly, SV40 was reported to induce telomerase activity in primary human mesothelial cells, but not in primary fibroblasts [151].

Human papillomavirus (HPV) is a small, double-stranded DNA virus that infects mucosal and cutaneous epithelial tissue [152]. The high-risk strains including HPV-8, -16, -18 and -31 cause malignant progression of lesions, whereas the low-risk strains including HPV-6 and -11 cause benign warts and lesions [153]. The E6 and E7 proteins encoded by “high-risk” strains including HPV-16 and -18 are oncoproteins that have been shown to have transformation properties [154]. When used in immortalization, E6 causes telomerase activation as well as accelerating the degradation of p53 by the 26S proteasome, whereas E7 inactivates Rb by preventing the binding of pRb to the E2F transcription factor [155, 156].

Human T-lymphotropic virus type 1 (HTLV-1) is the etiologic agent of adult T cell leukemia. Although HTLV-2 is less pathogenic than HTLV-1, both of the HTLV-1 and -2 Tax proteins, p40^tax^ (Tax1) and p37^tax^ (Tax2), share the capacity to immortalize lymphocytes in vitro [157]. HTLV-2 protein Tax2 demonstrates much stronger efficacy than that of Tax1 in immortalizing human T cells [158].

The *myc* oncogene family consists of several different members including *c*-*myc, N*-*myc, L*-*myc and B*- *myc*. *c*-*myc* expression is restricted to proliferating cells while *N*-*myc* and *L*-*myc* expression is associated with cellular differentiation [159, 160]. Moreover, the oncogene *myc* fulfils many of the expectations for a gene involved in immortalization of primary cells alone [160, 161] or cooperates with oncoproteins [162] or TERT [163].

B cell-specific Moloney murine leukemia virus integration site 1 (*BMI1*) which was identified as a c-*myc*-cooperating oncogene, is a critical transcriptional repressor for maintenance of proper gene expression during development [164–166]. INK4a locus, which encodes p16 and p19^Arf^, is an important target of* BMI1* and overexpression of* BMI1* extends replicative lifespan of human fibroblasts, probably through suppressing the p16-mediated senescence pathway [167, 168].

#### TERT

Telomerase is composed of two core components: the small nuclear ribonucleic acid (RNA) human telomerase RNA, which serves as an internal template for the synthesis of telomeric repeats, and the protein TERT (or hTERT in humans), which serves as a catalytic subunit that synthesizes the new telomeric DNA from the RNA template [22]. In most human primary cells, telomerase is either absent or present at an insufficient level for telomere maintenance [169]. TERT is the determinant for the presence of active telomerase [170, 171]. The introduction of ectopic expression of TERT is necessary for telomere-dependent senescence as it is able to significantly extend the lifespan of a variety of cell types, but it alone is not sufficient to immortalize them [172–174]. Theoretically, the abrogation of the Rb and p53 pathways with oncogenes or at a minimum, low p16 expression, is indispensable for cell immortalization [175, 176]. However, there are still investigations showing that TERT bypassed Rb and p53 pathway-dependent barriers to immortalize cells alone [176–180].

### Carcinogenic limitations and strategies

Despite the increasingly sophisticated strategies to immortalize human cells, there is still some debate over the risks upon integration of oncogenes into chromosomes. The primary safety concern with the use of a cell line is the transmission of an oncogenic factor to the host cells. Indeed, cells transduced with these oncogenes underwent additional changes including full carcinogenesis-associated changes (Table 2). The persistent infection by a subset of HPVs, especially HPV-16 and HPV-18, is etiologically linked to cervical cancer in women [181]. Deregulated overexpression of HPV E6 and E7 led to several alterations in cellular pathways and functions, which is associated with malignant transformation of cells and tumorigenesis [182]. In addition, HPV E6 oncoprotein can interact with hTERT to promote carcinogenesis in keratinocytes [183, 184].

**Table 2:** Malignant transformation and tumorigenesis during immortalization of primary cells

Immortalization	Cell type or animal		References
Oncoprotein(s)	Human	Biliary epithelial cells, fetal keratinocytes, fibroblasts, keratinocytes, mesothelial cells	[190, 346–349]
	Animal	Chinese hamster embryo fibroblasts, rabbit chondrocytes	[191, 350]
Oncoprotein(s) and oncogene(s)	Human	Colon smooth muscle cells, embryonic esophageal epithelial cell, epidermal keratinocytes, hepatocytes, primary fibroblasts, prostatic epithelial cells, mammary epithelial cells	[351–358]
TERT	Human	Astrocytes	[359]
TERT and viral oncoprotein(s)	Human	Airway (bronchial) epithelial cells, endothelial cells, esophageal epithelial cells, fibroblasts, hematopoietic progenitor cells, mammary epithelial cells, MSCs, ovarian surface epithelial cells	[202, 240, 360–368]
	Animal	Bovine adrenocortical cells	[369]
TERT and oncogenes	Human	Mammary epithelial cells, MSCs	[370, 371]
Oncoprotein(s), oncogene(s) and growth factors	Human	Oral keratinocytes	[372]
Oncoprotein(s) and chemical carcinogens	Human	Ectocervical and endocervical cells, oral keratinocytes	[373–377]
TERT, oncoprotein and alpha subunit of eukaryotic initiation factor 2	Human	Kidney cells	[378]

SV40 or SV40 sequences were found in several types of human cancers located in bone, brain, chest, etc. [185–188]. Evidence has shown that SV40 can successfully transform cell lines in vitro and induce tumors in neonatal hamsters in vivo [189–192]. The injection of SV40-transformed cells into terminally ill human patients caused subcutaneous tumor nodules [193]. Moreover, SV40-transduced cells contained integrated SV40 DNA, which was integrated at random positions on the cellular chromosomes of host cells [194, 195], leading to the controversial question of whether the virus poses a threat for further in vivo use.

Although introduction of hTERT is associated with fewer phenotypic and karyotypic changes of cells compared with SV40, the tumorigenicity of hTERT-transfected human cells remains controversial as well [176]. Previous studies have claimed that the hTERT-transduced primary human fetal lung fibroblasts, ameloblastoma cells and bovine mammary epithelial cells showed no malignant transformation [180, 196, 197]. Similarly, the hTERT-immortalized human MSCs past 290 population doublings showed no sign of malignant transformation or tumorigenesis in vitro and in vivo as the cells maintain contact inhibition and a stable protein expression profile as well as no tumor-like activity in immune-deficient mice [198–200]. Meanwhile, after subcutaneous injection with HPV-16 E6/E7 immortalized BMSCs into Nonobese Diabetic/Severe Combined Immunodeficiency (NOD/SCID) mice for 3 days, no tumor mass was observed compared to those injected with Hela cells in which tumor mass was observable [201]. Even the introduction of TERT and SV40 or HPV-16 E6/E7 was sufficient to immortalize ovarian surface epithelial cells and dermal papilla cells but not enough for tumor formation [179, 202–205].

However, other groups argue about the increased potential for tumor development in TERT-immortalized cells. On monolayer cultures, human MSCs and fibroblasts avoid cell-to-cell contact inhibition, anchor to culture dishes and tend to proliferate limitlessly [206, 207]. In clinical practice, elevated hTERT expression is a diagnostic marker for tumor and the overexpression of hTERT is claimed to be associated with an advanced invasive stage of tumor progression and poor prognosis [208–210]. Moreover, there are still concerns about genetic instability after TERT transfection or transduction. Spontaneous changes in *c*-*myc* proto-oncogene expression and other genetic alterations have been observed during in vitro culture of hTERT-immortalized human cells [211, 212].

Despite safety concerns for the immortalized cells, there are still some cases that successfully applied these cells for in vivo organ and tissue restoration. For liver impairment, Guo et al. [213] found that SV40-immortalized marmoset hepatic progenitor cells (MHPCs) injected into the injured liver of fumarylacetoacetate hydrolase-deficient mice repopulated with hepatocyte-like cells and MHPCs were also implanted as cholangiocytes into bile ducts of 3.5-diethoxycarbonyl-1,4-dihydrocollodine-induced bile ductular injured mice. Meanwhile, SV40-immortalized human fetal liver cells differentiated into mature hepatocytes after being transplanted into liver injured mice [214]. For brain damage, hTERT-immortalized cord blood MSCs were injected into the traumatically injured brain of a rat model and proliferated efficiently at the injury site for 2 weeks and showed no tumor formation in SCID mice after a 6-month observation [215].

To avoid persistent oncogene expression, conditional immortalization technology was developed. Conditional immortalization includes inserting a reagent mediate, operator controllable gene to create a cell line that can be expanded in a consistent fashion when the transgene is active. When desired clinical quantities of cell material are achieved, the transgene can be deactivated by the operator and the cells will return to a normal, post-mitotic state. The conditional immortalization technology c-MycER^TAM^ uses a combination of growth factor and 4-hydroxytamoxifen (4-HT) to activate the c-MycER transgene. In the absence of 4-HT, c-MycER is inactivated and the cells return to a normal phenotype [216]. Inactivation of SV40 LT was achieved using a temperature-sensitive mutant of the LT (SV40 tsA58) that is biologically active at permissive temperature (33.5 °C) but inactive at a non-permissive temperature (39 °C) [217]. Different vectors can have influence on the expression of transgenes. Unlike lentivirus, adenovirus does not integrate transgenes into the host genome and thereby can only provide a transient expression of the transgenes [218]. However, this kind of expression time is not controllable.

To acquire more accurate excision of oncogenes, site-specific recombination systems were developed. Cre/LoxP technology involves engineering a transgene flanked by LoxP sites. The transgene is activated until Cre recombinase is added. However, the cre-lox system is not 100% efficient and cells that have not deleted the transgene might require elimination [219, 220]. In addition, a Tet on/off system uses tetracycline responsive elements (TRE) that consist of a Tet operator and minimal promoter. The activation of the transgene and the subsequent cell division is related to tetracycline or doxycycline, which acts as a cue for activation (Tet-On) or inactivation (Tet-Off) [221, 222]. However, this technology still has the evident limitation termed “leakiness”, where the transgene continues to express at a low level even when the system is off [223]. Moreover, the transient activation of β-catenin was used to efficiently induce hTERT activation while silencing β-catenin suppresses the expression of hTERT [224]. Meanwhile, therapeutic strategies concerning transient activation of telomerase with small molecules, including the administration of 1% N-acetylcarnosine lubricant eye drops for prevention and treatment of cataracts, have been proven beneficial for dogs and other animals [225]. Huang et al. have identified that anthraquinone derivatives might be able to activate hTERT expression without causing genetic alterations in cells, whereas these cells fail to possess potent proliferative ability [226]. Interestingly, the introduction of some adenovirus derived genes, including the early 4 region (E4) of the adenoviral vector (*AdE4*), augments survival of human endothelial cells [227, 228]. However, investigations into *AdE4* gene products were largely overshadowed by the fact that these proteins not only orchestrated many viral processes, but also overlapped with oncogenic transformation of primary cells [229, 230]. Although creation of conditionally immortalized cell lines has the potential for therapeutic application, complete silence of the transgene before introduction into the patient’s body is still a concern that needs to be addressed.

### Potential loss of differentiation capacity

Differentiation capacity of MSCs and progenitor cells after immortalization is another concern that deserves more attention. A variety of reports has claimed that immortalization of progenitor cells will retain proliferative activity without compromising multipotent or specific differentiation potential of primary cells from species including human, mouse and porcine [231–237]. A similar phenomenon has been mentioned in human MSCs immortalized with SV40 [238, 239]. After serial transduction with hTERT, SV40 and H-Ras, human MSCs still retain their multilineage differentiation potential even during tumorigenesis [240, 241]. Moreover, Yang et al. [242] showed that hTERT-transduced human BMSCs seeded on porous polylactic glycolic acid (PLGA) scaffold have better osteogenic differentiation ability than primary human BMSCs seeded on scaffold. Similarly, human BMSCs immortalized with hTERT and HPV16 E6/E7 displayed greater differentiation potential far beyond the primary human BMSCs or even when human BMSCs expressed HPV-16 E6/E7 alone [243]. Interestingly, Okamoto et al. [244] reported that human BMSCs immortalized with hTERT and HPV-16 E6/E7 demonstrated significant clonal heterogeneity in differentiation potential. A similar phenomenon was found in mouse melanocyte progenitors that displayed distinct melanogenic differentiation potential [232]. More interestingly, there were opposite results as human primary dental pulp stem cells (DPSCs) are found to be approximately 60% more effective than hTERT-immortalized DPSCs in osteogenic differentiation [245]. For human placenta-derived MSCs immortalized with hTERT and BMI1, the differentiation potential was lost [246]. This discrepancy may partially be due to different immortalization strategies as the lost differentiation potential in ADSCs due to “SV40+hTERT” introduction can be preserved by “hTERT+BMI1” [247]. Moreover, cellular senescence counteracts the induction and reprogramming of induced pluripotent stem cells and senescence related INK4A/ARF and p53/p21 pathways are considered to be involved in these processes [248–251].

## Preconditioning of cells through matrix microenvironment optimization

Although genetic manipulation is a popular strategy for functional tissue engineering, it has limited clinical benefit due to its inherent risks [189, 193, 194]. For human cells that are sensitive to external changes, matrix microenvironmental alterations may modify intercellular communication, leading to enhanced proliferation ability without carcinogenic mutation [252, 253].

Cells in the body reside in a niche, a dynamic and complex environment, where extracellular cues provided allow cells to survive and maintain their balance between quiescence, self-renewal and differentiation [254, 255]. ECM, a versatile component that plays a key role in the stem cell niche, interacts with the resident cells by modulating cell behavior through its physical, biochemical and biomechanical properties [256, 257]. There is an increasing number of reports indicating that dECM is a promising substrate to maintain the stemness of expanded cells by mimicking the in vivo niche [258, 259]. dECM was found to improve the expansion capacity of human BMSCs [260], human and porcine SDSCs [261, 262], human umbilical cord MSCs [263] and porcine adipose stem cells derived from the infrapatellar fat pad [264]. Interestingly, dECM deposited by SDSCs could also rejuvenate somatic cells such as porcine nucleus pulposus cells [265, 266] and replicatively senescent porcine chondrocytes [267]. These dECM-expanded cells were smaller in size compared to those grown on plastic flasks. These results are in accordance with the finding from Whitefield and coworkers in which, under time-lapse video microscopy, the smaller cells were observed to continue proliferation, while the larger cells became senescent and exited the cell proliferation cycle [268].

Furthermore, current data indicate that dECM deposited by MSCs yields human adult SDSCs [269, 270] and porcine adipose stem cells derived from the infrapatellar fat pad [264] with better chondrogenic potential in vitro and with better repair capacity for cartilage defects in vivo [271]. Interestingly, BMSCs, a tissue-specific stem cell for endochondral bone formation, could be greatly recharged toward chondrogenic differentiation by expansion on dECM deposited by nonchondrogenic human urine stem cells [272] or human BMSCs themselves [260]. More interestingly, a recent report showed that, despite dECMs deposited by BMSCs and ADSCs enhancing the proliferation ability of MSCs, they failed to yield expansion of cancer cells (HeLa, MCF7 and MDA-MB-231) in terms of inhibiting the expansion ability of these cancer cells [273]. This finding indicates that normal cell derived dECM is not favorable for the growth of cancer cells. Given the undesirable potential of carcinogenesis after genetic modification from cell immortalization, dECM tends to be a better alternative.

In addition to rescuing replicative and differentiation capacities, dECM could reduce intracellular generation of ROS in aged murine BMSCs [274] and in human BMSCs [260] and umbilical cord MSCs [263]. Meanwhile, dECM could enhance the anti-oxidative capacity of human adult SDSCs [269] and protect umbilical cord MSCs from oxidative stress-induced premature senescence [275] to finally achieve better chondrogenic differentiation. Moreover, dECM could repress osteoclastogenesis in bone marrow monocytes through the attenuation of intracellular ROS [276]. All the above mentioned studies confirmed the anti-senescence and anti-oxidative effect of dECM as a culture substrate.

Mechanical cues, including stiffness and elasticity from the surrounding matrix microenvironment, are important cellular inputs that sustain cell proliferation and oppose cell senescence. Integrin-based focal adhesions are the main adhesion complex dominating mechanosensing [277]. In our previous study, dECM-expanded human BMSCs demonstrated increased expression of integrin α2 and β5 [260], which are potentially involved in the process of cell proliferation [278, 279]. As a powerful regulator of cell proliferation and survival, YAP/YAZ act as mechanotransducer that is regulated by F-actin cytoskeleton [280, 281]. Interestingly, dECM expansion was found to induce sustained activation of ERK1/2 as well as phosphorylated cyclin D1 human BMSCs [260] but decreased phosphorylated ERK in human adult source SDSCs [261]. A similar phenomenon was found in phosphorylated p38 expression in human SDSCs [261] and human umbilical cord MSCs [263] after dECM preconditioning. These discrepancies might be explained by the dual role of ERK [282] and p38 [283] signals that play upon cell senescence. The expression of Wnt5a and Wnt11a were also found to be upregulated following dECM expansion [261].

## Conclusions and perspective

Primary cells display a stable and long-term loss of proliferative capacity upon in vitro expansion despite continued viability and metabolic activity. This inability to proliferate is due to progressive shortening of telomeres during each replication which ultimately makes cells reach their “Hayflick limit”, termed telomere-dependent or replicative senescence. Meanwhile, there is another kind of senescence referred to as premature senescence since it can arrest cell growth long before reaching the “Hayflick limit”. One type is stress-induced senescence caused by the failure to simulate the in vivo supportive environment, which puts pressure on cell proliferation through the generation of ROS. In addition, both oncogenes and their counteracting tumor suppressors are proven to provoke premature senescence. A variety of signaling pathways are involved in all of these types of senescence, in which the p53/p21 and p16/Rb pathways are the two major signals involved. Oncogenes including SV40 and HPV-16 E6/E7 inhibit the p53/p21 and p16/Rb pathways, but are not able to immortalize primary cells unless followed by the introduction of TERT, which elongates telomeres, thereby abrogating the effect of the end replication problem. However, all of these genetic modification methods have the risk of virus introduction and potential oncogenesis, which must be addressed before its application into tissue engineering.

Optimization of the laboratory culture environment [259], including modulation of oxygen level and cell density and the introduction of growth factors, and recently discovered dECM preconditioning, is also an effective strategy to fight against senescence. Despite the fact that dECM deposited by MSCs from fetal [261] or young donors [272] offers a better rejuvenation effect in promoting aged MSCs in both expansion and differentiation capacities compared to adult donors, the source of these young cells was either allogenic or xenogeneic, which might pose a potential risk of compromising the donor [284] or immune rejection [285]. In this scenario, cells donated by the patients themselves are considered the best candidate. However, the elderly primarily suffer from degenerative disease and most of their autologous cells may suffer from senescence, which was identified as an influential factor in the quality of cells [286]. Given the demand for a younger cell population and the situation of carcinogenic transformation after genetic modification for immortalization purposes, it raises the possibility of combining genetic modification and environmental optimization strategies. In other words, we can immortalize these senescent cells and utilize their deposited dECM instead of the cells themselves to achieve a reduced senescent status and enhanced proliferation potential of expanded cells. The combination strategy might also overcome the potential loss of differentiation capacity of stem cells with the use of immortalization strategy alone. Further investigation into this matrix microenvironmental preconditioning-based rejuvenation strategy may offer important insights into possible means of providing robust primary cells as therapeutic agents.

## Abbreviations

4-HT: 4-hydroxytamoxifen
ADSCs: adipose-derived stromal cells
AKT: protein kinase B
ATM: ataxia-telangiectasia mutated
BMI1: B cell-specific Moloney murine leukemia virus integration site 1
BMP: bone morphogenetic protein
BMSCs: bone marrow-derived MSCs
BTG3: B cell translocation gene 3
CKIs: cyclin-dependent kinase inhibitors
DDR: DNA damage response
dECM: decellularized cell-deposited extracellular matrix
DNA: deoxyribonucleic acid
DPSCs: dental pulp stem cells
ECM: extracellular matrix
ERK1/2: extracellular signal-regulated kinase 1/2
FGF: fibroblast growth factor
H_2_O_2_: hydrogen peroxide
HIF: hypoxia inducible factor
HPV: human papillomavirus
HTLV-1: human T-lymphotropic virus type 1
IGF: insulin-like growth factor
JNK: c-Jun N-terminal kinase
LATS1: large tumor suppressor 1
LRP5: low-density lipoprotein receptor-related protein 5
MAPK: extracellular signal-regulated kinase
MEK: extracellular signal-regulated kinase kinase
MSCs: mesenchymal stem cells
mTOR: mammalian target of rapamycin
NF1: neurofibromatosis 1
OH^−^: hydroxyl radical
O_2_^−^: superoxide anion
PI3Ks: phosphatidylinostitide 3 kinases
PLGA: polylactic glycolic acid
PTEN: phosphatase and tensin homolog
RNA: nuclear ribonucleic acid
Rb: retinoblastoma protein
ROS: reactive oxygen species
SASP: senescence-associated secretory phenotype
SDSCs: synovium-derived stem cell
SV40: simian virus 40
SV40 LT: SV40 large T
SV40 ST: SV40 small t antigen
TERT: telomerase reverse transcriptase
TGFβ: transforming growth factor β
TRE: tetracycline responsive elements
VHL: von Hippel-Lindau tumor suppressor
Wnt: Wingless/IntMDM2: mouse double minute-2 homolog
YAP: yes-associated protein 1
